# Outcomes of autologous bone marrow mononuclear cell administration in the treatment of neurologic sequelae in children with spina bifida

**DOI:** 10.1186/s13287-023-03349-w

**Published:** 2023-04-28

**Authors:** Liem Thanh Nguyen, Huong Thu Le, Kien Trung Nguyen, Hang Thi Bui, Anh Phuong Thi Nguyen, Doan Van Ngo, Duc Minh Hoang, Minh Duy Ngo

**Affiliations:** 1 Vinmec Research Institute of Stem Cell and Gene Technology, Vinmec Healthcare System, 458 Minh Khai, Hanoi, Vietnam; 2 College of Health Science, VinUniversity, Vinhomes Ocean Park, Gia Lam District, Hanoi, Vietnam; 3 Vinmec International Hospital – Times City, Vinmec Health Care System, 458 Minh Khai, Hanoi, Vietnam

**Keywords:** Spina bifida, Neurological sequelae, Autologous bone marrow mononuclear cell

## Abstract

**Background:**

To evaluate the safety and efficacy of autologous bone marrow mononuclear cell (BMMNC) infusion in the management of neurological sequelae in children with spina bifida (SB).

**Methods:**

BMMNCs were harvested from bilateral anterior iliac crests. Two intrathecal BMMNC administrations were performed with an interval of 6 months. The measurements of outcomes included clinical assessments, cystomanometry and rectomanometry.

**Results:**

Eleven children with SB underwent autologous BMMNC infusions from 2016 to 2020. There were no severe adverse events during the study period. The number of patients requiring assistance to expel stools decreased from 11 before cell infusion to 3 after the second cell infusion. The number of patients who had urine leakage decreased from 9 patients at baseline to 3 patients after the second BMMNC infusion. The mean bladder capacity increased from 127.7 ± 59.2 ml at baseline to 136.3 ± 54.8 ml at six months and to 158.3 ± 56.2 ml at 12 months after BMMNC infusions. Detrusor pressure (pdet) decreased from 32.4 ± 22.0 cm H_2_O at baseline to 21.9 ± 11.8 cm H_2_O after 12 months of follow-up. At baseline, six patients could walk independently. After the 2nd infusion, eight patients could walk independently.

**Conclusion:**

Intrathecal infusions of autologous bone marrow mononuclear cells are safe and may improve bowel, bladder, and motor function in children with SB.

*Trial registration*: NCT, NCT05472428. Registered July 25, 2022- Retrospectively registered, https://www.clinicaltrials.gov/ct2/show/NCT05472428.

**Supplementary Information:**

The online version contains supplementary material available at 10.1186/s13287-023-03349-w.

## Background

Spina bifida (SB) is one of the most common and severe malformations of neural tube defects, with two types, including SB aperta and SB occulta, and different subtypes (myeloschisis, myelomeningocele, meningocele, lipomeningocele, and spinal dorsal dermal sinus tract) [1]. The prevalence of SB varies according to geographic area and time, from 3.5 per 10,000 live births in the United States to 6.25/10,000 live births in China and 13/10,000 in Africa [2]. The spinal cord abnormalities seen in children with SB have been attributed to congenital myelodysplasia, as well as to its exposure to amniotic fluid and postpartum local trauma [3]. These injuries result in severe neurological sequelae. A study by Freeman KA et al. with 3252 patients showed that only 40.8% of participants achieved urinary continence, and 43% achieved fecal continence [4]. Chronic renal failure is a consequence of urinary dysfunction in long-term follow-up. Among 427,616 spina bifida hospital admissions with a mean age of 26 years, 35,249 patients (8%) suffered from chronic renal insufficiency [5]. In addition, Sileo et al. demonstrated that only 40% of infants can walk independently or require minor support [6].

The quality of life of children with SB and their caregivers is severely affected. Children with fecal incontinence have many difficulties travelling and socializing. SB has a severe negative impact on parents’ psychological well-being. Parents face many challenges associated with their child’s disability [7, 8]. The prevalence of depressive symptoms in parents with SB children was up to 48% [9], while 36.3% of adults with SB require antidepressants for the purpose of treating depressive symptoms [10]. Prenatal repair has been investigated in many centers to improve the long-term outcomes of children with SB. This approach could reduce the need for shunting and improve motor outcomes but could increase both maternal and fetal morbidity [11]. There are different conventional treatments for fecal and urinary incontinence due to spina bifida sequelae such as clean intermittent catheterization, biofeedback, electric stimulations, laxative medicaments, and retrograde colon enemas, but they do not improve these conditions[12].

Recently, cellular therapy has been a treatment option for a variety of neurological conditions, such as spinal cord injury, brain stroke, brain trauma, and cerebral palsy, with promising results [13–19]. Cellular therapy has also been intensively investigated for experimental SB in fetal animal models. In 2008, Fauza et al. reported the results of neural stem cell injection into the spinal cord in an ovine model of fetal surgery for spina bifida. They found that the procedure was safe. The highest density of neural stem cells was detected in the most damaged areas of the spinal cord. They retain an undifferentiated state and produce neurotrophic factors within the defect [20]. In 2015, Dionigi et al. demonstrated that simple intra-amniotic injection of concentrated amniotic mesenchymal stem cells could induce partial or complete coverage of experimental spina bifida. They also reported that transamniotic stem cell therapy minimized Chiari-II malformation in experimental spina bifida [21]. In 2019, Shieh et al. showed that transamniotic injection of amniotic mesenchymal stem cells or placental mesenchymal stem cells can induce partial coverage of experimental spina bifida in a leporine model [22]. Bone marrow-derived mesenchymal stem cells (BM-MSCs) were also applied for fetal experimental SB with promising outcomes. When injecting human bone marrow-derived mesenchymal stem cells, human neural stem cells, or human foreskin fibroblasts into the amniotic cavity of chick embryos with spinal open neural tube defects, Lee et al. showed that the best reclosure was observed in the BM-MSC group [23]. Li et al. transplanted a chitosan–gelatin scaffold seeded with BM-MSCs in rat fetuses with retinoic acid-induced spina bifida aperta. They found that prenatal transplantation of the scaffold combined with BM-MSCs could decrease the defective area of the spinal cord, and the skin defect was almost closed in many fetuses [24]. In 2019, Boruczkowski et al. published a study using Wharton’s jelly stem cell administration for children with SB. They demonstrated that Wharton’s jelly stem cell administration was safe and improved motor functions, micturition/defecation control, and cognitive functions in children with SB [25]. The aim of this study was to evaluate the safety and efficacy of BMMNC infusion in improving bowel, urinary and motor functions in 11 children with SB.

## Methods

### Study design

An open-label, uncontrolled phase I clinical trial was performed.

### Research setting and duration

The study was carried out at Vinmec Times City International Hospital from July 2016 to December 2020.

### Patients

The patients were enrolled in the study in compliance with the inclusion and exclusion criteria as presented below.

#### Inclusion criteria

- The patient who was diagnosed with lumbar spina bifida underwent spinal cord close-up surgery.
- Both genders.
- Aged between 6 months and 15 years old.
- Exhibited bowel disorders (constipation, fecal incontinence) and urinary dysfunction (urinary retention or leakage).

#### Exclusion criteria

- Vertebrae clefts in the chest, neck, and other spinal locations.
- Coagulopathy.
- Acute and chronic infection.
- Kidney function disorder, liver failure
- Patients with complex cardiovascular diseases (including valvular heart disease, cardiomyopathy, arrhythmia, congenital heart disease, and hypertrophy syndrome).
- Distress.

### Sample size

Eleven patients with spina bifida who met these criteria were included in the study.

### Bone marrow-derived mononuclear cell procedure

#### Bone marrow aspiration and preparation

Bone marrow was bilaterally aspirated from anterior iliac crests under general anesthesia in an operating theater at Vinmec International Hospital. The required bone marrow volume was calculated in accordance with each participant’s body weight. Based on our prior experience, this volume was determined as follows: 8 mL/kg for patients who weighed less than 10 kg and [80 mL + (body weight in kg—10) × 7 mL] for patients who weighed more than 10 kg, with a total volume of no more than 200 mL [26].

Mononuclear cells and autologous plasma were isolated from the aspirated bone marrow by gradient centrifugation using Ficoll-Paque (GE Healthcare, Sweden) in a cleanroom following the ISO 14644 standard at Vinmec Research Institute of Stem Cell and Gene Technology. The BM-MNCs were washed two times with phosphate-buffered saline and adjusted in 10 mL normal saline for administration. The release criteria for BM-MNCs were as follows: endotoxin < 5.0 EU/mL (measured by Endosafe, Charles River, Wilmington, Massachusetts), more than 80% viability using 7-AAD evaluation, and mycoplasma negative (MycoAlert PLUS *Mycoplasma* detection kit, Lonza, Switzerland). Additional quality control included bacterial and fungal contamination (the BacT/Alert three-dimensional microbial detection system, Biomerieux, USA), mesenchymal stem cell marker evaluation (human MSC analysis kit, Becton and Dickinson company, USA), and complete blood count analysis of aspirated bone marrow and collected BMNCs.

### Intervention

Two BMMNC administrations were performed with an interval of 6 months. An 18-gauge spinal cord was inserted intrathecally through a space between the 4th and 5th lumbar vertebrae under general anesthesia by an experienced pediatric anesthetist. Three milliliters of CSF was allowed to drop out spontaneously, and then 10 ml of normal saline containing BMMNCs was infused through this needle over the course of 30 min using an electric pump.

### Outcome measures

#### Procedure-related adverse events

The number of adverse events (AEs) and severe adverse events (SAEs) during BMMNC administration and during 72 h of hospital stay at the first and second administrations, and 12 months after discharge were monitored. SAEs were defined as death, any cardiac event (new ventricular tachycardia, ventricular fibrillation, asystole, cardiac arrest, cardiac hypertrophy, heart attack, etc.), acute pulmonary distress and pulmonary embolism, stroke, anaphylactic shock, sepsis, thrombosis, and acute inflammatory response. The AEs included fever, common allergic reaction, infection at bone marrow aspiration and administration sites, changes in vital signs, and abnormal laboratory test results.

### Efficacy

Urinary function was assessed via clinical manifestations such as bladder sensation, urinary retention, and urinary incontinence. In addition, cystomanometry was performed to evaluate urinary function, including bladder compliance, bladder capacity, and maximum detrusor pressure (pdet-max). Voiding cystourethrograms were carried out to evaluate bladder morphology and vesicoureteral reflux.

Bowel function was assessed via clinical manifestations such as constipation based on the Bristol stool scale [27], Rome criteria IV [28] and fecal incontinence. In addition, anorectal manometry was performed to assess rectal pressure, anal pressure, rectoanal inhibitory reflex (RAIR), first sensation, and urge of defecation.

Lower limb motor function was assessed via clinical manifestations and manual muscle testing (MMT) [29].

### Ethics statement

The study protocol was reviewed and approved by the Ethics Committee of the National Pediatric Hospital with approval number 1193/BVNTU-VNCSKTE and Decision of the Ministry of Health No. 612/QD-BYT allowing stem cell infusion to treat spinal cord injury. This study was registered at ClinicalTrials.gov (number NCT05472428).

### Statistical analysis

Descriptive statistics were used to illustrate the demographics of the spina bifida patients expressed as the means with standard deviations or medians with ranges for quantitative data.

## Results

### BMMNC isolation and quality control

**Fig. 1:**
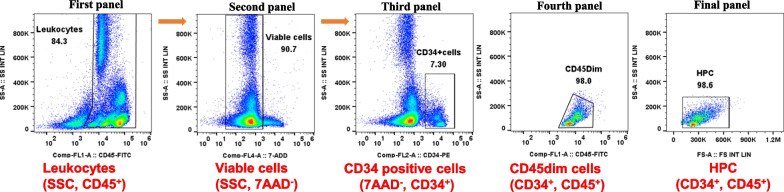
Representative images of the gating strategy for the CD34+ population and cell viability using 7-AAD staining. Initially, leukocytes were gated using CD45+ to exclude platelets, red blood cell debris, and aggregates (first panel). Subsequently, viable CD45+ cells were identified as 7-AAD negative (second panel), followed by the identification of the CD34+ population (third panel). The fourth panel displays CD45 dim-positive cells, and the final panel indicates the identification of the true hematopoietic stem cell population (CD34+, CD45+)

Bone marrow aspiration and BMMNC isolation were successfully carried out in all patients. The total BMMNCs isolated from the 1st and 2nd administrations were (839 ± 86.37) × 10^6^ cells and (959.41 ± 121.55) × 10^6^ cells, respectively. The total CD34+ cell counts were (65.96 ± 5.61) × 10^6^ cells for the first administration and (63.49 ± 10.46) × 10^6^ cells for the second, with viability higher than 95% in both administrations. The percentage of MSCs present in the BMMNC population was 0.083% ± 0.02% (all, mean ± SEM, *n* = 11). Details are provided in the sheet (Cell) of the Additional file 1 and Fig. 1.

### Patient characterization

Between 2016 and 2020, 11 patients who met all inclusion and exclusion criteria were included in this study, with an average age of 4.5 ± 2.8 years (mean ± SD, *n* = 11) and a sex ratio of 45.4% vs. 54.5% (male vs. female). All 11 patients required support for defecation, whereas 10 children aged over 1 year old were unable to feel the urge of defecation or urination at baseline. Ten patients required clean intermittent catheterization (CIC), and one patient had frequent urinary leakage.

At baseline, anorectomanometry revealed that the resting, squeeze, cough, and strain anal pressures were 36.2 ± 25.6, 39.9 ± 15.5, 44.7 ± 11.0, and 35.8 ± 11.4 mmHg, respectively. The rectal pressure at rest, squeeze, cough, and strain was −1.3 ± 8.4, 22.5 ± 15.7, 25.3 ± 9.0, and 8.5 ± 2.8 mmHg, respectively (Table 1).

**Table 1:** Anorectal manometry results before and after cell infusions

	Baseline (*n* = 6)*	After the 2nd infusion (*n* = 6)*
Rectal pressure (mmHg), mean ± SD		
Rest	−1.3 ± 8.4	−8.0 ± 14.2
Squeeze	22.5 ± 15.7	17.1 ± 5.1
Cough	25.3 ± 9.0	104.0 ± 0.0
Strain	8.5 ± 2.8	15.0 ± 4.3
Anal pressure (mmHg), mean ± SD		
Rest	36.2 ± 25.6	43.6 ± 23.1
Squeeze	39.9 ± 15.5	38.4 ± 19.4
Cough	44.7 ± 11.0	70.6 ± 67.7
Strain	35.8 ± 11.4	43.9 ± 30.3
RAIR (mL), mean ± SD	21.7 ± 4.1	16.7 ± 8.2
The first sensation (mL), mean ± SD	42.5 ± 5.0	33.3 ± 11.5

*2 patients < 2 years old at baseline, 3 patients whose parents refused the procedure

Cystometry revealed that the maximum detrusor pressure (pdet max) was 32.4 cm H_2_O, and the bladder volume was 127.7 mL (mean ± SD) at baseline (Table 2).

**Table 2:** Cystometry results before and after cell infusions

	Baseline (*n* = 7)	After the 1st infusion (*n* = 7)	After the 2nd infusion (*n* = 7)
Bladder capacity, mean(ml)	127.7 ± 59.2	136.3 ± 54.8	158.3 ± 56.2
Pdet max – mean (cmH_2_0)	32.4 ± 22.0	24.4 ± 20.4	21.9 ± 11.8

### Complications and side effects

No severe adverse events (SAEs) occurred during the study period. At the end of the trial, seven AEs were confirmed to be possibly related to BMMNC infusion, including headache (three events), vomiting (one event), fever (one event), urethralgia (one event), and swelling at the bone marrow extraction site (one event). All adverse events resolved spontaneously (see details in the safety sheet of the Additional file 1).

### Bowel function

All eleven patients required completed support for stool evacuation (enema, abdominal compression, or anal stool extraction) at baseline. This number was reduced significantly to 6 out of 11 patients after the first infusion and 3 out of 11 patients after the second infusion.

The defecation urge feeling could be assessed in seven patients aged 3 and over. The results indicated that the number of patients increased from none at baseline to 5 patients after the first BMMNC infusion and 6 patients after the second BMMNC infusion.

A significant improvement was observed in the Bristol stool scale, in which the number of patients with Bristol type I decreased from 10 patients at baseline to 3 patients and 2 patients after the first and the second BMMNC infusion, respectively. The number of patients who had constipation via ROME IV decreased from 11 patients at baseline to six patients after the second BMMNC infusion.

Anorectal manometry could be performed in 6 patients. The results showed that the RAIR decreased from 21.7 ± 4.1 ml to 16.7 ± 8.2 ml, and the first sensation decreased from 42.5 ± 5.0 at baseline to 33.3 ± 11.5 ml after the second BMMNC infusion (Table 1).

### Bladder function

Bladder sensation could be evaluated in seven patients aged 3 and over. Urinate urge feelings were only noted in 1 patient at baseline, but they increased to 3 after the first infusion and 4 after the second infusion.

Ten out of 11 patients had to use clean intermittent catheterization (CIC) at baseline, and this figure did not change after BMMNC infusions.

The number of patients who had urine leakage decreased from 9 patients at baseline to 3 patients after the second BMMNC infusion (1 patient had urinary retention, and 1 patient was not assessed due to being under the age of 12 months).

**Fig. 2:**
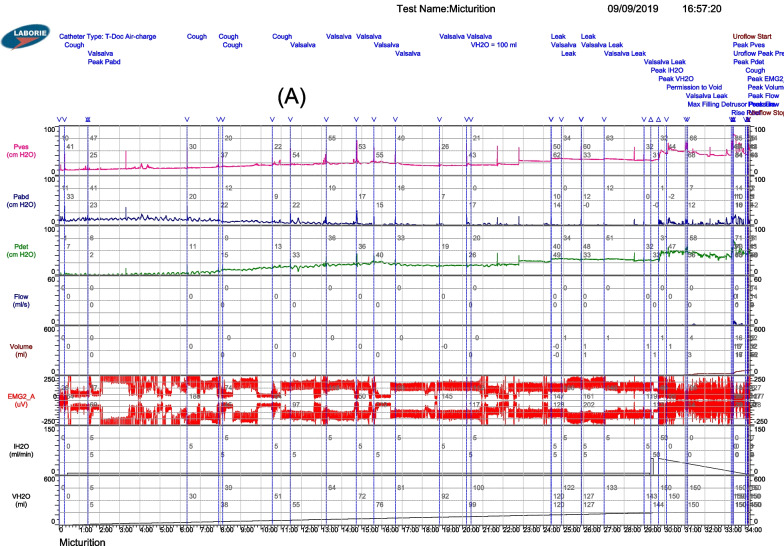

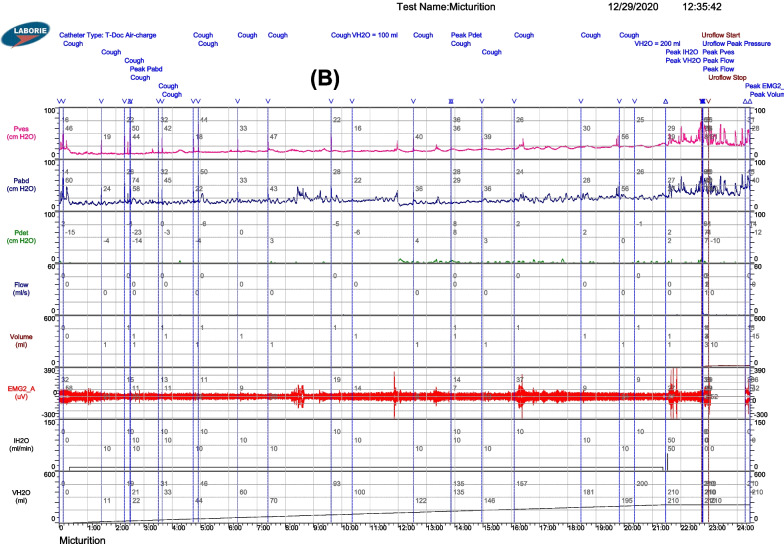
Cystometry measurements were taken to assess bladder capacity, compliance, and detrusor pressure in patient code 11. The results indicated poor bladder compliance, low bladder capacity, and high pdet max at baseline (**A**). However, after 12 months of follow-up, there was a notable improvement in bladder pressure, with a decreased pdet max value, better bladder compliance, and an increased bladder capacity (98% vs. 86%) following stem cell transplantation (**B**). These findings suggest that stem cell transplantation may be a promising treatment option for patients with bladder dysfunction

Cystomanometry was carried out in 7 patients. The mean bladder capacity increased from 127.7 ± 59.2 ml at baseline to 136.3 ± 54.8 ml at six months and to 158.3 ± 56.2 ml at 12 months after BMMNC infusions. Pdet decreased from 32.4 ± 22.0 cmH_2_O at baseline to 21.9 ± 11.8 cmH_2_O after 12 months of follow-up. (Table 2, Fig. 2).

**Fig. 3:**
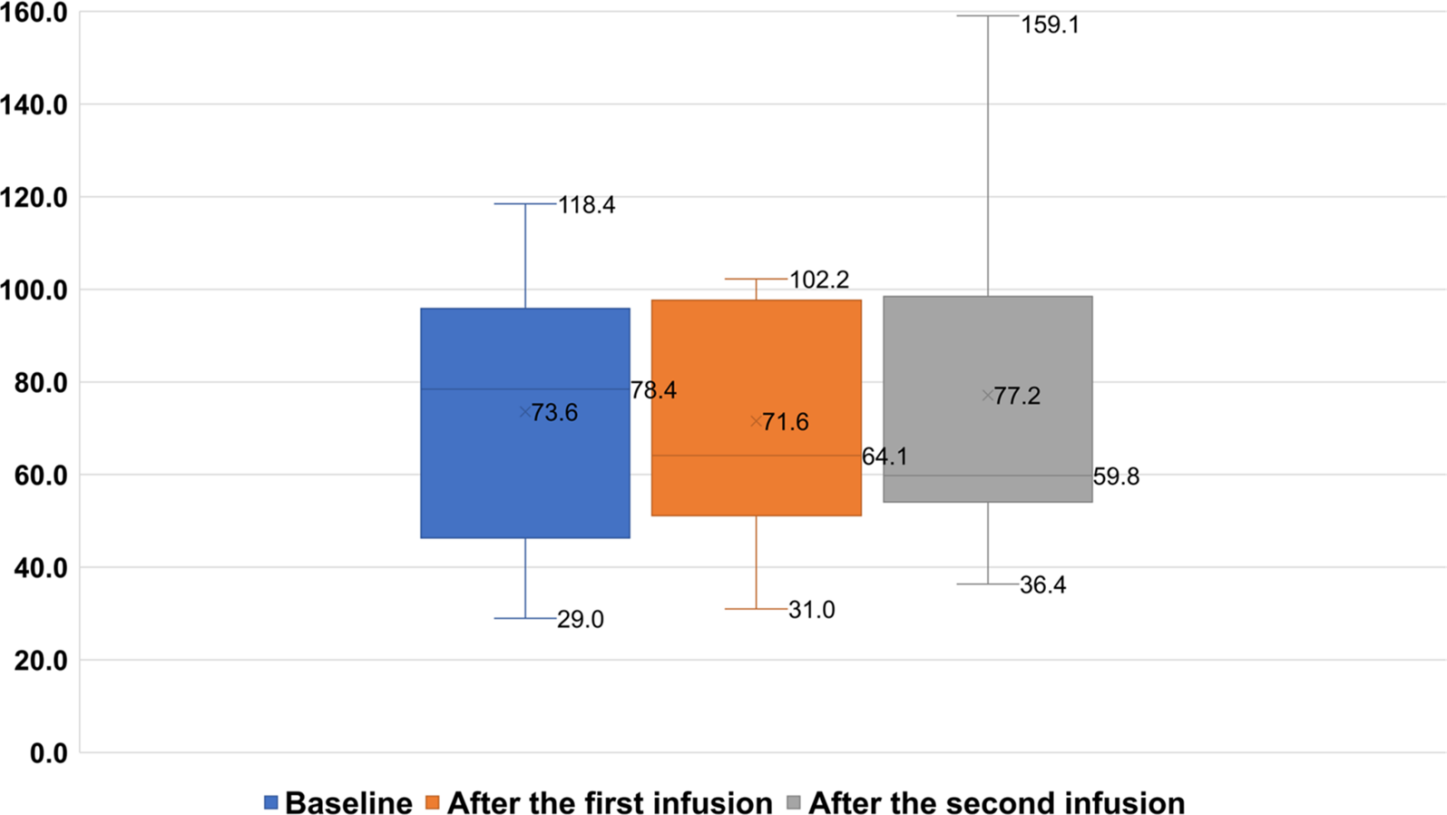
The percentage of bladder capacity over time (%), with the age factor removed. The median percentage of bladder capacity to bladder capacity with the age factor removed improved

The median percentage of bladder capacity with the age factor removed decreased from 78.4 (46.3–95.8) (%) at baseline to 64.1 (51.1–97.6) (%) and 59.8 (54.1–98.4) (%) after the first and second infusion, respectively (Fig. 3).

**Fig. 4:**
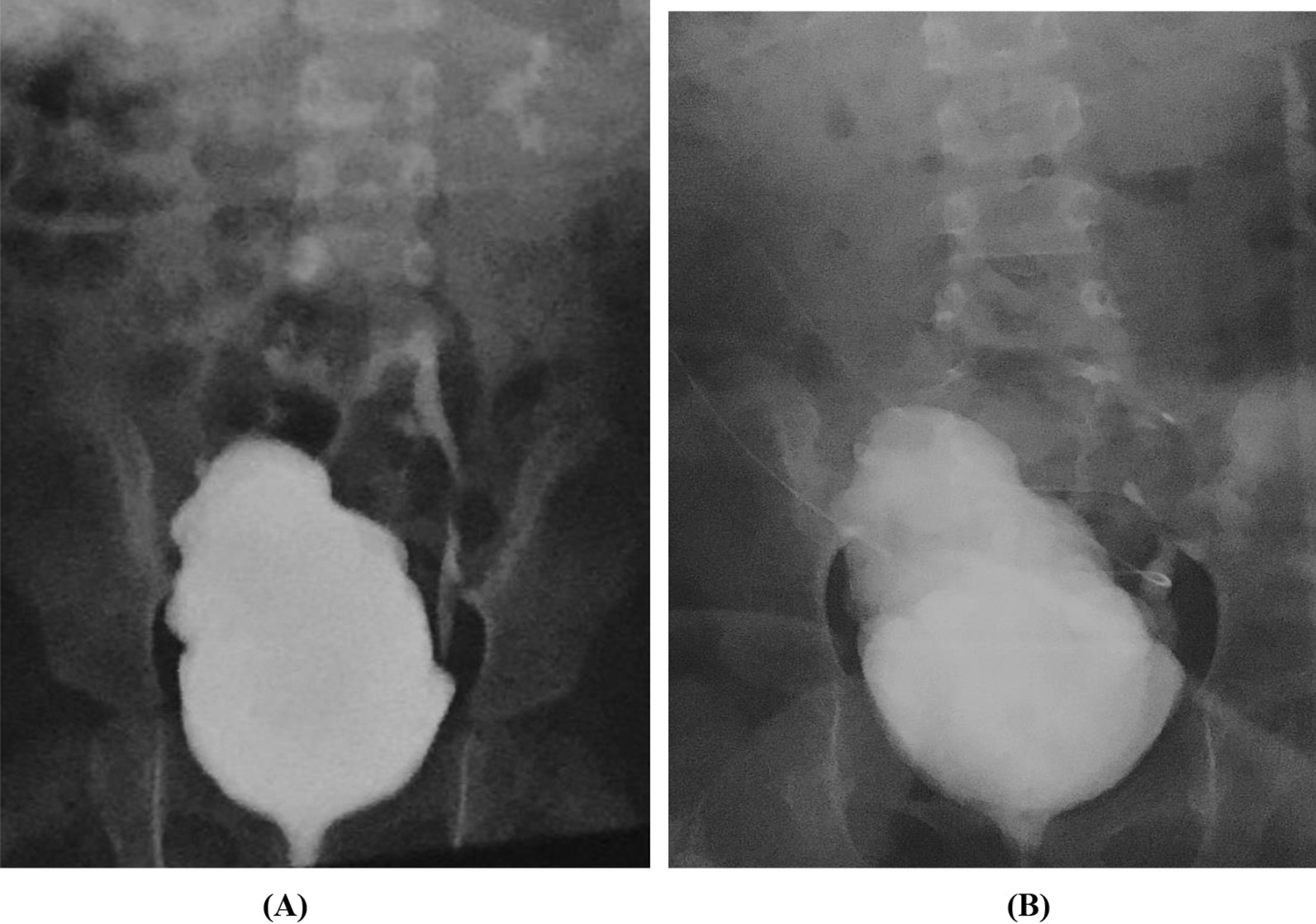
The resolution of left vesicoureteral reflux in Patient 4 following stem cell infusions. At baseline, the patient presented with grade III left vesicoureteral reflux (**A**), but after 12 months of stem cell infusion therapy, there were no images of left vesicoureteral reflux (**B**), indicating a successful treatment outcome. These findings suggest that stem cell therapy may be a promising treatment option for vesicoureteral reflux

A voiding cystourethrogram was performed in 10 patients. Abnormalities in the bladder walls were observed in 6 patients, and vesicoureteral reflux was noted in 2 patients. After 12 months of follow-up, the rate of abnormal bladder images remained unchanged. The vesicoureteral reflux disappeared in 1 patient and persisted in another (Fig. 4).

### Lower extremity motor function

At baseline, 6 patients could walk independently, 3 patients needed ankle foot orthotic (AFO) support, 1 patient had a stiff ankle joint, and 1 patient could not be assessed due to being under the age of 12 months. After the 2nd infusion, 8 patients could walk independently, 2 patients needed AFO support, and 1 patient had a stiff ankle joint. The patient who could not be assessed (under 12 months at baseline) could walk independently after the second infusion.

Manual muscle testing (MMT) could be performed at baseline in 9 patients. The number of patients who had normal lower limb muscle strength (MMT = 5) increased from 2 patients at baseline to 3 patients after BMMNC infusions.

Two patients could not be assessed with MMT due to being under 3 years of age. However, two patients could walk independently after the second infusion (Table 3).

**Table 3:** Lower extremity motor function before and after cell infusions

	Baseline (*n* = 11)	After the 2nd infusion (*n* = 11)
Walk independently	6	8
AFO	3	2
Stiff ankle joint	1	1
Aged under 12 months	1	0

## Discussion

The results of our study indicate that bone marrow aspiration from anterior iliac crests and BM-MNC administration via the intrathecal route are safe for patients with spina bifida. The bone marrow harvest was feasible and safe to perform in all 11 patients. There were no severe adverse events, including intrathecal bleeding, and impairment of the cardiovascular system, lung, kidneys, or neurological systems, during hospitalization or within the 12-month follow-up. The safety of the intrathecal route was also reported by other studies. In children, this route has been safe for delivering chemotherapy compounds in children with cancer, injecting baclofen in the treatment of cerebral palsy, performing regional anesthesia in surgery and realizing pain relief post-surgery to infuse cells for patients with cerebral palsy, autism, etc. [13–16, 30–38]. Our study supports previous studies regarding the safety of intrathecal administration in children, as we did not observe any severe adverse events throughout the course of our study. We chose the intrathecal route to deliver cells because the cells will be able to reach the medullary directly compared to intravenous administration, where most of the cells are trapped in the lung or spleen [39].

The results of our study demonstrated that the neurological sequelae of spina bifida were improved after autologous BMMNC infusions. Bowel function was significantly improved after cell therapy. The number of patients who were able to defecate without assistance increased from zero at baseline to five patients after the first cell infusion and eight after the second cell infusion. Two patients did not have improved bowel function. In addition, positive changes were also noted on anorectal manometry.

Regarding urinary function, although all patients did not urinate voluntarily after cell infusion, bladder function was significantly improved according to cystomanometry. In a previous study, Neveus et al. demonstrated that the normally expected bladder capacity up to the age of 12 is calculated as (age + 1) × 30 mL, with the bladder capacity in healthy children ranging from 65–150% [40]. Although bladder capacity in our study increased, the percentage of bladder capacity with the age factor removed decreased after two infusions with a median (range) value at baseline of 78.4 (46.3–95.8), decreasing to 64.1 (51.1–97.6) after the first infusion and 59.8 (54.1–98.4) after the second infusion. In addition, pdet decreased significantly after cell administration. These changes contribute to protecting renal function because they can prevent or lessen the severity of vesicoureteral reflux. During the 12-month follow-up period, the number of patients with vesicoureteral reflux did not increase in 10 patients, whereas the reflux disappeared in 1 patient who had vesicoureteral reflux at baseline.

Improvements have also been observed in motor function. The number of patients who could walk independently increased from 6 at baseline to 8 after two cell infusions. Our results are in accordance with previous studies. Sharma et al. showed that there were no complications in 13 patients with SB who were injected with bone marrow mononuclear cells into the spinal cord caudal space [41]. Boruczkowski et al. reported that administration of mesenchymal stem cells for children with SB was safe and ameliorated motor function and micturition/defecation control [25].

To elucidate the mechanism of cell therapy for spina bifida, a great deal of research has been conducted in animal models. Li et al. demonstrated that transplanted mesenchymal stem cells in the spinal cord of rats with induced spina bifida could survive, grow, and express markers of neurons, glia and myoblasts. In addition, transplanted MSCs could also reduce spinal tissue apoptosis [42, 43]. Ma et al. indicated that the transplantation of MSCs into the spinal cord of rats could promote the transplanted MSCs and the surrounding cells to differentiate into a sensory neuron cell fate and protect sensory neurons [44]. It was illustrated that administration of CD34+ cells in combination with MSCs enhanced putative blood vessel formation and peripheral nerve growth in spina bifida patients, which, in turn, promoted the improvement of bladder function [45]. In 2021, Kunpalin et al. performed a systematic review and meta‐analysis of 26 published studies using cell administration in utero for spina bifida in animal models. The results from this study revealed that intra‐amniotic injection of allogeneic amniotic fluid mesenchymal stem cells is safe and effective in covering myelomeningocele defects in small animals, while transplantation of human placenta mesenchymal stem cells to the spinal cord of fetal lambs is safe and effective in enhancing lower limb motor function [46]. All of the above animal studies showed that BM-MNCs/MSCs could repair motor and sensory deficiencies by differentiating into neural lineage cells, secreting growth factors, and proliferating blood vessels.

## Conclusions

In conclusion, our study demonstrated that BMMNC infusion into the medullary cavity is safe and can ameliorate bowel, bladder, and motor function in children with SB. However, a randomized clinical trial with a larger sample should be performed in the future to obtain an accurate conclusion.

## Supplementary Information

**Additional file 1.** The supplementary database comprises seven sheets of information on eleven patients, including monitoring milestones, adverse events, cell data, results from anorectal manometry, bladder and cystometry tests, and MMT assessments.

## Data Availability

All data generated or analysed during this study are included in this published article and its Additional files.

## Abbreviations

AEs: Adverse events
BM-MSCs: Bone marrow-derived mesenchymal stem cells
BMMNC: Bone marrow mononuclear cell
CIC: Clean intermittent catheterization
MMT: Manual muscle testing
Pdet-max: Maximum detrusor pressure
RAIR: Rectoanal inhibitory reflex
SAE: Severe adverse events
SB: Spina bifida
